# Loss of A20 in BM-MSCs regulates the Th17/Treg balance in Rheumatoid Arthritis

**DOI:** 10.1038/s41598-017-18693-0

**Published:** 2018-01-11

**Authors:** Zhuan Feng, Yue Zhai, Zhaohui Zheng, Lijie Yang, Xing Luo, Xiwen Dong, Qing Han, Jin Jin, Zhi-Nan Chen, Ping Zhu

**Affiliations:** 1 Department of Clinical Immunology, Xijing Hospital, The Fourth Military Medical University, No. 127 West Changle Road, Xi’an, Shaanxi Province People’s Republic of China; 2 Department of Cell Biology, Fourth Military Medical University, Xi’an, China; 3 National Translational Science Center for Molecular Medicine, Xi’an, 710032 China; 4 Department of hematology, Xijing Hospital, The Fourth Military Medical University, No. 127 West Changle Road, Xi’an, Shaanxi Province People’s Republic of China

## Abstract

Mesenchymal stem cells (MSCs) are multi-potent cells that are self-renewable and possess the potential to differentiate into multiple lineages. Several studies demonstrated that MSCs could regulate a Th17/Treg balance and could be a potential therapeutic target for Rheumatoid Arthritis (RA). A20 is highly expressed in many cell types after the stimulation of TNF-α, where it may inhibit pro-inflammatory cytokine secretion. However, the expression of A20 in BM-MSCs in RA is not fully understood. In our study, we found that A20 was decreased in RA patients’ bone marrow MSCs (BM-MSCs), and with more IL-6 secretion, the balance of Th17/Treg was broken. In CIA mice, we found a moderate A20 decrease in mice MSCs as compared with those of control group in mRNA and protein levels. However, the IL-6 expression was increased. After umbilical cord MSCs treatment, A20 and IL-6 expressions were equal to the control group. Thus, our study indicates that loss of A20 in MSCs regulates the Th17/Treg balance in RA and the regulatory role of A20 in pro-inflammatory IL-6 production could be a potential target for the transfer of MSCs in RA adoptive therapy.

## Introduction

Rheumatoid arthritis (RA) is a chronic autoimmune inflammatory disease that has an incidence of 0.5% to 1%^1^, with high incidence in women and the elderly^2^. In the past two decades, the biologics and small-molecule kinase inhibitors are effective methods to treat RA^3^, but it is limited by potential long-term toxicity. The climax of clinical stage is articular inflammation, the reversal of excessive immune response is the main therapeutic target to improve physical function and life quality^4^.

Mesenchymal stem cells (MSCs) are multi-potent stem cells from mesoderm, which were separated by Friedenstenin firstly^5^, and widely exist in various tissues of human body, such as amniotic membrane, umbilical cord blood, blood vessel, cartilage, placenta and etc. MSCs possess self-renew and multi-directional differentiation potency^6^, could differentiate into different types of cells^7^, and regulate series of immunological responses such as effecting immune responses between T and B cells, inhibiting T cell proliferation, dendritic cell maturation, and NK cell activation^7^. As a result of its low level of MHC II molecule, MSCs have low immunogenicity which enable the adoptive transfer of MSCs from different species to realize injection therapy^8^. It has been reported that the capacity of MSCs to reduce disease burden is largely associated with their ability to modulate the activity of the host immune responses rather than to contributing directly to tissue regeneration^9^. As a matter of fact, systemic infusion of human adipose-derived mesenchymal stem cells significantly decreased the severity of arthritis, ameliorated the symptoms, and prevented joint damage in CIA mice^10^. Besides, treatment of DMARDs with UC-MSCs injection to articular cavity could provide safe, significant, and persistent clinical benefits for patients with active RA who are nonresponsive to classical medications^11^. Several clinical experiments have verified the therapeutic effect of MSCs. A non-randomized comparative clinical trial with RA patients who were unresponsive to classical medications^11^, refers to that the function of MSCs treatment is correlate with increased numbers of Treg cells in peripheral blood, but the exact cause is misty. These studies indicate MSCs have huge perspective in treating RA, but deep research is needed.

A20, also called TNF-α induced protein 3 (TNFAIP3), was firstly reported in 1990, which was induced by primary responsive gene stimulated by TNF-α in epithelial cell^12^. It contains two ubiquitin-editing domains. The amino terminal domain of A20 removes lysine-63 (K63)-linked ubiquitin chains from receptor interacting protein (RIP), an essential mediator of the proximal TNF receptor 1 (TNFR1) signaling complex. The carboxy-terminal domain of A20, composed of seven C2/C2 zinc fingers, targets RIP for proteasomal degradation^13^. The anti-inflammation function of A20 has been well documented. For example, using mice with thymus-specific deletion of A20, Catrysse *et al*.^14^ showed that A20 was a major cyto-protective protein in the development of chronic joint inflammation. Genome-wide association studies (GWAS) found that A20 was susceptible to many self-immune diseases, like RA, systemic lupus erythematosus (SLE), inflammatory bowel disease (IBD) and so on. Many studies indicate that A20 was a negative regulation protein to anti-inflammatory reaction through inhibiting NF-κB pathway^15–19^. The expression of A20 by immune cells, such as dendritic cells (DCs) and macrophages, maintains immune homeostasis and prevents autoimmune diseases. A20 is required for the termination of TNF-induced signals, through which A20 perform an anti-inflammatory role by shutting down the production of downstream inflammatory cytokines^20^. However, little is known about the function of A20 in MSCs in RA.

Therefore, we carry out this experiment to investigate the possible role of MSC A20 expression in RA development, and determine the underlying mechanism to seek for potential RA therapeutic target.

## Results

### A20 was decreased in RA patients’ BM-MSCs

**Figure 1:**
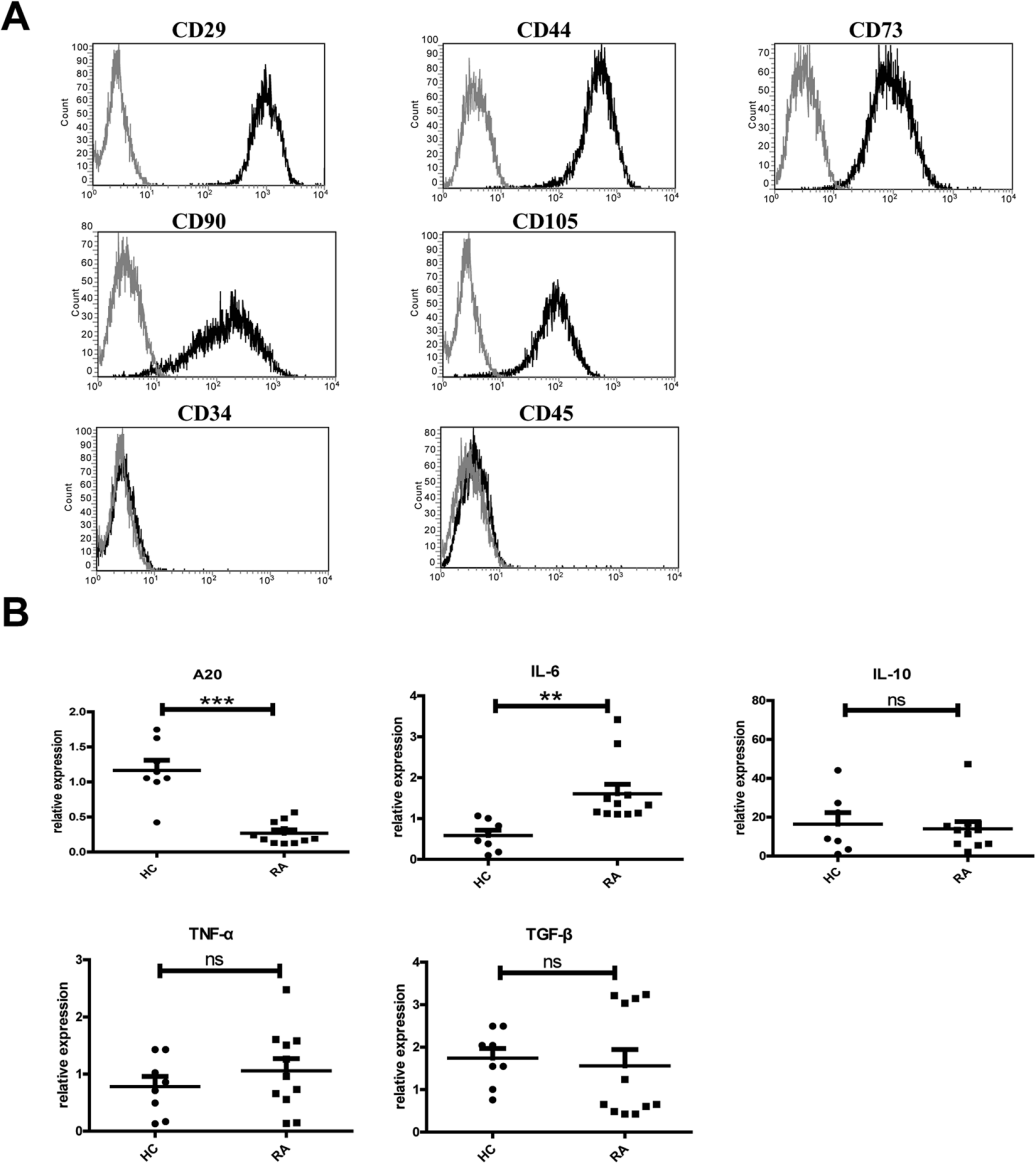
A20 expression was down-regulated in BM-MSCs of RA patients. (**A**) Cell surface markers were analyzed by flow cytometry. BM-MSCs were uniformly positive for the MSC markers CD29, CD44, CD73, CD90, and CD105; and negative for CD34 and CD45; (**B**) A20, IL-6, IL-10, TNF-α, and TGF-β mRNA levels were examined by RT-PCR analysis.

According to the International Society for Cellular Therapy (ISCT) criteria^21^, BM-MSCs were analyzed by flow cytometry based on MSC markers uniformly positive for the CD29, CD44, CD73, CD90, and CD105, and negative for CD34 and CD45 (Fig. 1A). Expression of A20 was significantly decreased in RA (n = 11) BM-MSCs samples compared with those of HCs’ (n = 8). Meanwhile, expression of the inflammatory cytokine IL-6 in MSCs was evidently increased in RA patients than HCs. Other cytokines, namely IL-10, TNF-α and TGF-β remain unchanged (Fig. 1B).

### TNF-α induces A20 expression in UC-MSCs

**Figure 2:**
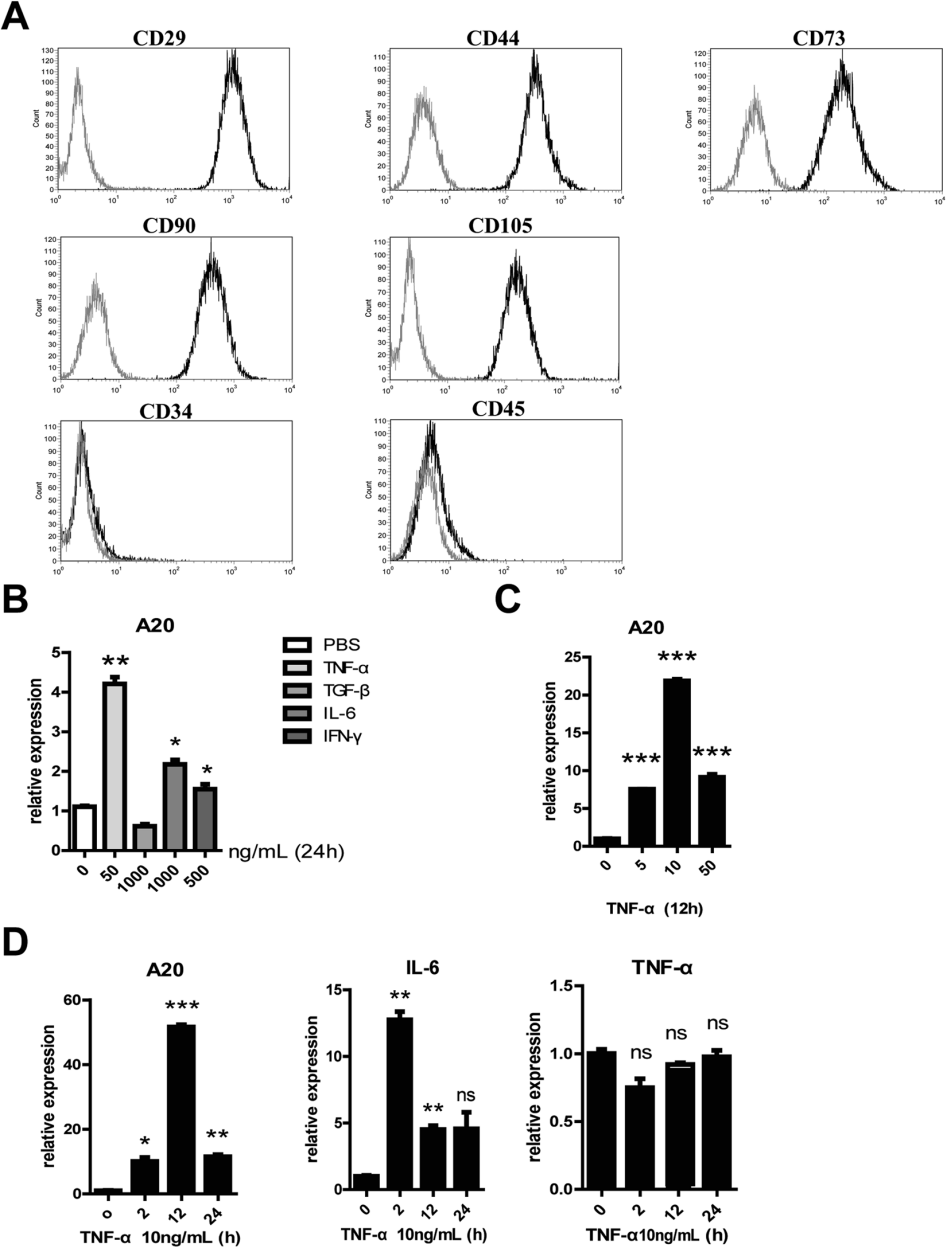
Changes in the expression of A20 and IL-6 in UC-MSCs stimulated by TNF-α. (**A**) Cell surface markers were analyzed by flow cytometry. UC-MSCs were uniformly positive for the MSC markers CD29, CD44, CD73, CD90, and CD105, and negative for CD34 and CD45; (**B**) A20 transcription has been shown to be up-regulated in TNF-α, IL-6, and IFN-γ stimulation significantly; (**C**) UC-MSCs were treated with 0, 5, 10, and 50 ng/mL TNF-α for 12 hrs. (**D**) A20, IL-6, and TNF-α mRNA levels were examined by RT-PCR analysis. UC-MSCs were treated with 10 ng/mL TNF-α for 0, 2, 12, and 24 hrs, and mRNA expression level was determined by RT-PCR analysis. ***p < 0.001, **p < 0.01, *p < 0.05.

Since human umbilical cord MSCs (UC-MSCs) that are easy to enrich share similarities with BM-MSCs in immunogenicity and therapeutic function, UC-MSCs have been suggested as an alternative source of MSCs for cell therapy^22^. UC-MSCs and BM-MSCs expressed similar MSC membrane markers (Fig. 2A). The expression of A20 could be induced by various stimuli^12^. However, which inflammatory stimuli could induce the expression of A20 in UC-MSCs haven’t been identified. We therefore detected A20’s expression after TNF-α, TGF-β, IFN-γ and IL-6 treatment. RT-PCR showed that A20 increased significantly by TNF-α, IL-6 and IFN-γ treatment (Fig. 2B). In detail, the expression of A20 induced by TNF-α elevated in a dose and time-dependent manner, peaking at 10 ng/mL after 12 hours treatment (Fig. 2C, D). And A20 transcription has been shown to be up-regulated in IL-6 and IFN-γ treatment besides TNF-α stimuli, but they don’t have a dose-dependent effect (Fig. S2). IL-6 changed contrarily, which was rapidly expressed after TNF-α treatment for 2 hours, while significantly decreased at 12 hours. Expression of TNF-α remains unchanged throughout (Fig. 2D).

### A20 plays an anti-inflammatory role in UC-MSCs

**Figure 3:**
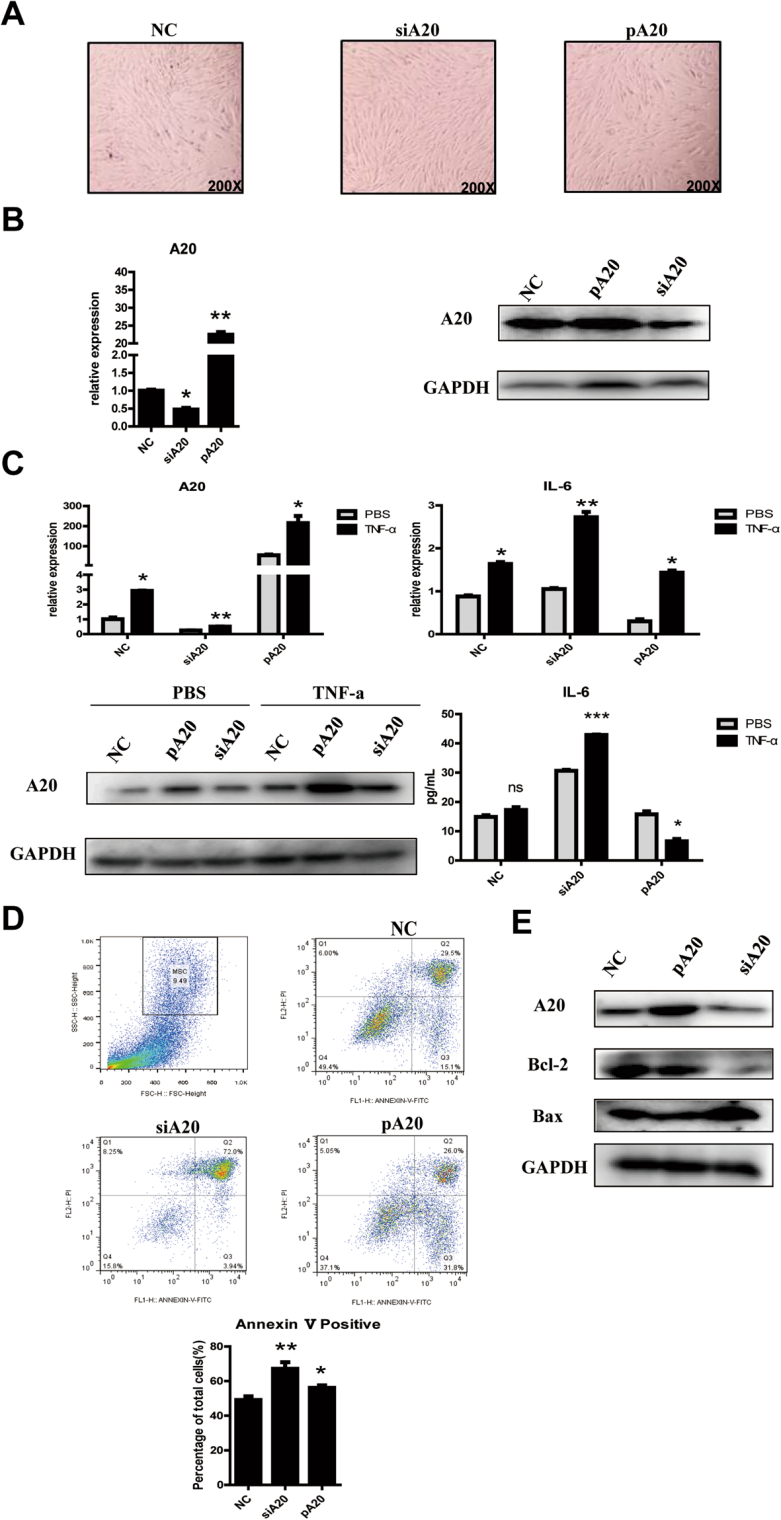
A20 overexpression inhibited IL-6 to attenuate inflammation response. (**A**) Morphological characterization of UC-MSCs after transfected siA20 and overexpression A20 plasmid. (**B**) RT-PCR and Western blots were performed to determine the expression levels of A20 mRNA and protein. The relative expression was determined by normalization to GAPDH. Results were obtained from three independent experiments. (**C**) RT-PCR, Western blots and Elisa analyses of A20, IL-6 levels with A20 down-regulation or overexpression at the stimulation of 10 ng/mL TNF-α for 12 hrs. (**D**) Flow cytometry examined apoptosis of UC-MSCs with or without A20. (**E**) Western blots were performed to determine the expression levels of Bcl-2 and Bax proteins. Western blots displayed are cropped images. Full-length blots are presented in Supplementary Figure 1. ***p < 0.001, **p < 0.01, *p < 0.05. All experiments were repeated three times.

To further investigate immunosuppressive capacity of A20, we then constructed A20 knock-down and over-expression system in UC-MSCs. Therefore, A20 short-interfering RNA (siRNA) and a A20 overexpression plasmid were transducted to UC-MSCs respectively. The RT-PCR and western blot assays confirmed successfully knock-down or overexpression of A20. All three groups namely control UC-MSC, siA20 UC-MSC and pA20 UC-MSC, exhibited strong adherence and classical spindle-shaped morphology of MSCs in optical microscope (Fig. 3A). As depicted in Fig. 3B, we successfully overexpressed or down-regulated A20 mRNA and protein in UC-MSCs. When A20 was interfered, IL-6 was higher than the two other groups. When A20 was overexpressed, the expression of IL-6 mRNA and protein were reversed with or without TNF-α stimulus (Fig. 3C). Compared with control group, siA20 group had higher apoptosis rate (Fig. 3D). Beyond that, we also detected Bcl-2 and Bax proteins showing siA20 group had higher apoptosis rate than the control group (Fig. 3E).

### A20 regulates Th17/Treg balance in UC-MSCs

**Figure 4:**
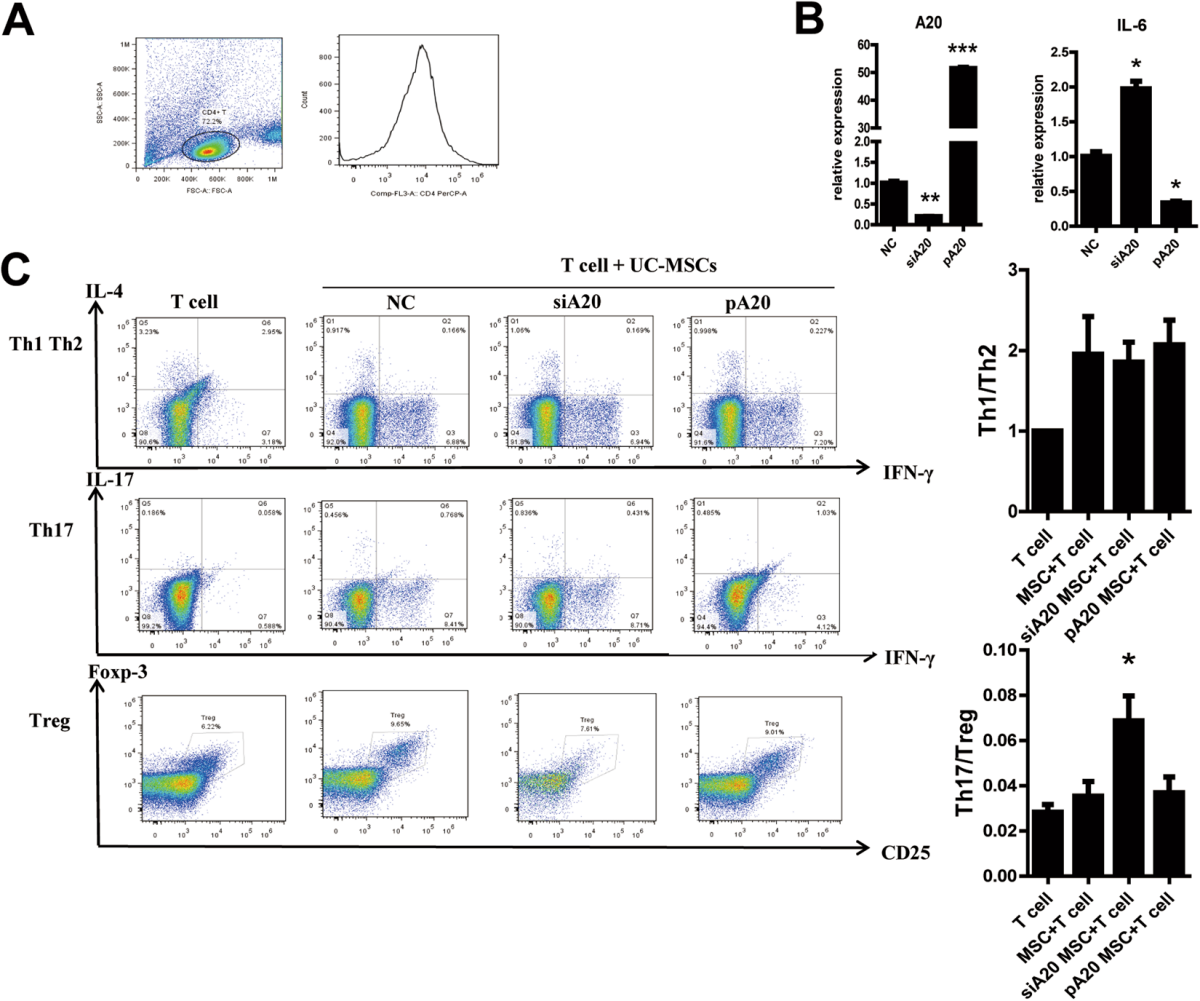
A20 overexpression in UC-MSCs increased the ratio of Th17/Treg co-cultured with CD4^+^ T cell by inhibiting IL-6. (**A**) CD4^+^ T cell isolated from PBMC was cultured alone or co-cultured with control UC-MSCs, siA20 UC-MSCs and pA20 UC-MSCs. Cells were subjected to flow cytometry. (**B**) After co-culture, we detected A20 and IL-6 expressions in control UC-MSCs, siA20 UC-MSCs, pA20 UC-MSCs. (**C**) We also measured the ratio of Th1/Th2 and Th17/Treg, the results showed that Th17/Treg was up-regulated in siA20 UC-MSCs significantly, but Th1/Th2 had no great change. ***p < 0.001, **p < 0.01, *p < 0.05. All experiments were repeated three times.

To determine whether changes of A20 in UC-MSCs would affect the differentiation of CD4^+^ T cells, we co-cultured UC-MSCs with CD4^+^ T cell isolated from healthy individuals’ peripheral blood mononuclear cell (PBMC) (Fig. 4A). After co-culture, we detected A20 and IL-6 expressions in control UC-MSC, siA20 UC-MSC, pA20 UC-MSC. IL-6 expression level changed contrary to A20 level (Fig. 4B). Th1, Th2, Th17 and Treg cells were labeled as IFN-γ^+^, anti-IL-4^+^, anti-IL-17^+^ and anti-Foxp3^+^ respectively. After co-culture with siA20 UC-MSCs, the ratio of Th17/Treg has a significant increase. While the differentiation of Th1 and Th2 were not affected (Fig. 4C). Meanwhile, IL-6 was significantly increased in siA20 group (Fig. 4B).

### CIA mice have low expression of A20 than control mice in BM-MSCs

**Figure 5:**
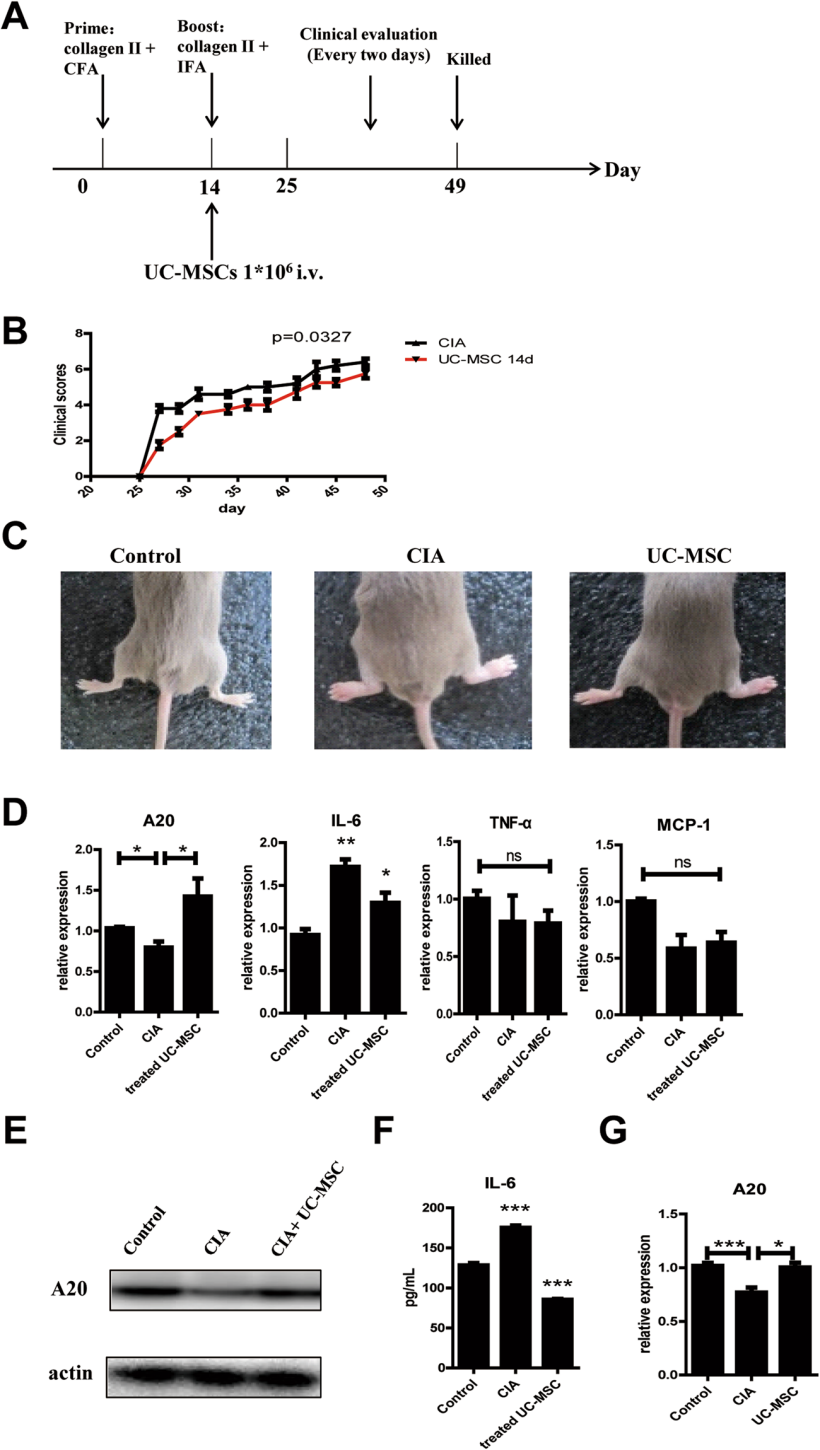
A20 expression was down-regulated in BM-MSCs of CIA mice. (**A**) Schematic shows the immune injection procedures. Arrows indicate the days when the UC-MSCs injections were given. Clinical joint scores (**B**) after therapeutic treatment with 1 * 10^6^ i.v. UC-MSCs treatment. (**C**) Photographs showing the paw swelling in control, CIA or UC-MSCs treated mice (n = 5/group). (**D**) RT-PCR analysis of A20, IL-6, TNF-α, and MCP-1 mRNA levels on UC-MSCs treatment day 14. (**E, F**) Western-blot analysis of A20 and ELISA analysis of mice serum IL-6 level. Western-blots displayed are cropped images. Full-length blots are presented in Supplementary Figure 1. (**G**) The expression of A20 in UC-MSCs before treatment and in BM-MSCs in CIA mice. ***p < 0.001, **p < 0.01, *p < 0.05 as determined by the Mann-Whitney test.

CIA mice model were induced by two steps of immunization and examined every two days after 27 days of first immunization (Fig. 5A). After immunization of incomplete Freund’ adjuvant (IFA), ten out of fifteen mice developed CIA with obvious swelling toe and limited mobility lasting for 3–4 weeks. Normal controls did not show joint swelling, the UC-MSCs treatment group had significant degree of disease remission (Fig. 5B). The paws after the induction of RA are shown in photographs, UC-MSCs treatment markedly inhibited the edema of the arthritic joints compared with CIA mice (Fig. 5C). The expression of A20 in BM-MSCs was lower in CIA mice compared with control group. Level of IL-6 was elevated at the same time, other pro-inflammatory mediators including TNF-α and MCP-1 hadn’t changed significantly (Fig. 5D). After UC-MSCs treatment, arthritis swelling and limited mobility was improved. A20 expression level returned normal (Fig. 5D, E). At the same time, IL-6 expression also decreased into a similar level to control group in serum (Fig. 5F). Mice BM-MSCs A20 level in adoptive UC-MSCs treatment group was significantly higher than CIA group (Fig. 5G). These results indicate MSCs expressed high A20 could therapy CIA in some extent, so it can be a considering criteria in cell therapy in the future.

## Discussion

Mesenchymal stem cells (MSC) are multi-potent cells from mesoderm that are self-renewable and possess the potential to differentiate into multiple lineages. The function of MSCs has been documented before; such as MSC-mediated immune-suppression decreased tumor growth^23^, and attenuated inflammatory activities. Many molecules are reported to have played a role in the immune-regulation process in MSCs, for example, CD25 and IL-35. MSCs suppress CD25 mRNA translation by regulating the LKB1-AMPK-mTOR pathway to potentiate T-cell suppression and over-express IL-35 of MSCs can effectively inhibit proliferation and function of CD4^+^ T cells^24–27^. According to the reports, human gingiva-derived MSCs could significantly inhibit Th17 cells and simultaneously promote the expansion of Treg cells in acquired immune system^28^. In mice, human gingiva-derived MSCs control the development and severity of CIA depending on Treg cells, and transplantation of gingiva-derived MSCs can efficiently ameliorate severity of the CIA inflammation by T cell homeostasis and Fas/FasL^29,30^. However, few studies focused on A20 in BM-MSCs. In our research, we found that A20 inhibit NF-κB signal pathway activation which attenuates the expression of pro-inflammatory cytokine IL-6 in MSCs. Apart from the inhibition of pro-inflammatory cytokines, A20 also promote the induction of anti-inflammatory cytokines such as IL-10 and etc^23^. All these results verified a negative regulatory role of A20 in inflammation.

The most exciting result we found here was that A20 was decreased in RA patients’ BM-MSCs compared to control group. Considering A20 is up-regulated through NF-κB activation and then plays a negative feedback role in NF-κB signaling pathway, the loss of A20 expression in BM-MSC is able to cause severe pro-inflammatory cytokine secretion. For one thing, in RA, the levels of TNF, IL-1 and IL-6 in serum and synovial fluid are increased^31,32^, which give a profitable environment for NF-κB activation, so that loss of A20 expression could be one of the important causes of continuous immune response in RA. For another, different from other immunocytes, MSCs mainly serve as immune-suppression cells in autoimmune diseases, and the abnormal immune activation of MSCs would induce more severe inflammation. Based on the above considerations, our findings may have important implications for understanding the cause of the RA.

Our results also demonstrated that UC-MSCs treatment could increase MSCs A20 expression so that was able to inhibit arthritis and reduce the expression of pro-inflammatory cytokine (especially IL-6) in CIA mice. Unlike previous research that mainly focus on the immunologic changes induced by direct contact of MSCs with other immune or no immune cells, we put more emphasis on the expression of cytokines that are critical component of local immune micro-environment and are responsible for differentiation of immune cells. For example, IL-6 is a key pro-inflammatory cytokine that promotes the differentiation of pro-arthritis Th17 cells, blocks the generation of anti-inflammatory Treg cells and triggers systemic inflammatory processes, such as infiltration of inflammatory cells^33^. Such results could also be verified in our research, as the increased expression of IL-6 induced by interfering A20 could significantly reduce Treg cells and increase Th17 cells. Therefore, in our research we confirmed that A20 could negatively regulate the expression of IL-6 and that overexpression of A20 could reverse IL-6 induced Th17 up-regulation.

Apart from a negative regulator of NF-κB related immune activation signaling pathway, A20 harbors an anti-apoptotic potentiality. Impairment in immuno-modulatory function of MSCs (for example, induction of apoptosis) may also affect the pathogenesis of RA. Due to insufficient apoptosis the production of inflammatory mediators are the pathological hallmarks of RA^34^. A20-regulated caspase-8 activity and inflammatory response mediated in RIPK1-dependent manner may play an important role in auto-inflammatory diseases. A20 might provide an important mechanism to control the activation of RIPK1 and caspase-8. The kinase activity of RIPK1 is critical not only for necroptosis, but also for RIPK1-dependent apoptosis that mediates the activation of caspase-8 and inflammation^35,36^. In endothelial cells from TNF-, Fas-, and NK-mediated cell death, A20 targets the TNF apoptotic pathway by binding the protease domain of caspase-8 and inhibits its dimerization and p-MLKL activation^36–39^. Beyond that, in renal cell, A20 binds to ASK1 and promotes the degradation of ASK1 leading to the suppression of JNK activation and the blockade of apoptosis^40,41^. Whether the anti-apoptotic role of A20 participated in the immune-regulatory exertion of MSCs remains to be elucidated. As the beginning research of the role of A20 in MSCs as potential therapeutic target in RA, we did not dig further in this aspect, which deserves further experiments in the future. Besides, a main flaw of our research is that the expression of A20 or cytokines is mainly detected by RT-PCR and ELISA rather than Western Blot. However, it seems justifiable as the cell numbers of RA patients’ BM-MSCs are too little for detection by Western Blot, and that the RT-PCR and ELISA results are potent enough for the results.

In conclusion, we found that A20 expression was down-regulated in both human RA patients and CIA mice BM-MSCs. Overexpression of A20 inhibited the expression of IL-6 in UC-MSCs that could reduce excessive expression of IL-6 and further restore Th17/Treg balance which may be a potential target for RA.

## Materials and Methods

### Patients and BM-MSCs acquisition

Eleven RA patients and eight healthy controls (HCs) were recruited in the experiments (informed consent had been obtained). All RA patients met the diagnostic criteria of the American College of Rheumatology^42^. The ethics approval for the human part of this study was granted by Ethical Committee of Fourth Military Medical University, and all experimental protocols were carried out in accordance with the relevant guidelines and regulations. The median age of the RA patients was 45 years, ranging from 35 to 70 year (Table 1). BM-MSCs were obtained from the respective RA patients during bone marrow aspiration biopsy in Xijing Hospital (Xi’an, China). Disease characteristics from all subjects were summarized in Table 1. After bone marrow aspiration biopsy, BM-MSCs were separated by density gradient centrifugation. The cells were seeded in culture flasks with DMEM-HG supplemented with 10% FBS (Gibco, Grand Island, USA), 2 mmol/L L-glutamine and 50 U/mL penicillin and streptomycin (both from Lonza). Cell cultures were maintained at 37 °C in a 5% CO_2_ humidified atmosphere. Non-adherent cells were removed when the medium was changed after 2–3 days.

**Table 1:** Characteristics of rheumatoid arthritis (RA) patients and healthy controls (HC).

Characteristics	HC	RA
Number of patients	8	11
Age in years, median (IQR)	36.0 (32.0–50.0)	50 (40.0–61.0)
Female sex, (%)	6 (75)	9 (81.8)
Disease duration, month, median (IQR)	na	8.5 (1.25–14)
Anti-CCP positive, IU/ml median	na	<25
ESR, mm/hour, median (IQR)	na	33 (11.6–73.0)
CRP, mg/dL, median (IQR)	na	6.42 (0.793–8.83)
DAS28, median (IQR)	na	8 (2.6–9)

Values are presented as median (interquartile range) or number (percentage). IQR, interquartile range, Anti-CCP, anticyclic citrullinated peptide antibodies; ESR, erythrocyte sedimentation rate; CRP, C-reactive protein; DAS28, 28-joint disease activity score; na, not applicable; compared to RA patients.

### Mice

All experimental procedures applied to mice were approved by Ethical Committee of Fourth Military Medical University, and were performed in compliance with the national regulations. DBA mice were purchased from the Beijing Vital River Laboratory Animal Technology Co., Ltd. (Beijing, China), and were maintained in a specific pathogen-free mouse facility (room temperature, 20–22 °C; room humidity, 40–60%, with free access to food and water) under a 12 hrs light/dark cycle. CIA was induced by injecting DBA/1 with bovine type II collagen (CII) emulsified in complete Freund’s adjuvant (CFA), followed by boosting 14 days later with CII emulsified in IFA and treated with UC-MSCs. The development of arthritis was monitored until day 49 and the arthritis scores were evaluated every two days. All procedures were performed with the approval of the Committee on the Use of Live Animals in the Fourth Military Medical University. CIA model of DBA mice was induced by injection of bovine type collagen in IFA on day 0 and day 14. Inflammation was evaluated by the following scale: 0 = no damage; 1 = paw with detectable swelling in a single digit; 2 = paw with swelling in more than one digit; 3 = paw with swelling of all digits and instep; and 4 = severe swelling of the paw and ankle. DBA/1 mice with collagen II-induced arthritis were treated with UC-MSCs from human. At the moment of the boost (day 14), UC-MSCs were infused (1 × 10^6^ cells) into CIA mice (n = 5) via the lateral tail vein. In the control group, mice received phosphate buffered saline (PBS) infusion (n = 5). DBA mice were monitored twice weekly for signs of arthritis based on paw swelling and arthritis scores.

### Real-Time Quantitative PCR assay

For RT-PCR analysis, total RNA was isolated by E.Z.N.A. Total RNA Kit II (OMEGA, BioTek, Norcross, USA) in RNase-free conditions and lysed in RNA lysis buffer from MSCs with then reverse transcribed into cDNA with Superscript first strand synthesis kit (Invitrogen) according to the manufacture’s protocol. RT-PCR was then performed with a SYBR Green PCR kit (TaKaRa, Otsu, Japan) on the Mxpro system to determine the expression levels of the genes of interest. All primers were synthesized by The Beijing Genomics Institute (Shenzhen, China). The sequences of PCR primers are listed in Table 2.

**Table 2:** Primer Sequences for Real-Time Quantitative PCR.

Real-time quantitative PCR Mouse primers.	GAPDH	forward 5′-AGGTCGGTGTGAACGGATTT-3′
		reverse 5′-GGGGTCGTTGATGGCAACA-3′
	MCP-1	forward 5′-GCATCTGCCCTAAGGTCTTC-3′
		reverse 5′-TGCTTGAGGTGGTTGTGGAA-3′
	IL-6	forward 5′-GATGCCCCAGGCAGAGAA-3′
		reverse 5′-CACCCAGGGAATTCAAATGC-3′
	TNF-α	forward 5′-GGAAACCCAGACGCATTGA-3′
		reverse 5′-TCAGGATCTGGCCCTTGAAC-3′
	A20	forward 5′-AAACCAATGGTGATGGAAA-3′
		reverse 5′-GTTGTCCCATTCGTCATTCC-3′
Real-time quantitative PCR Human primers	GAPDH	forward 5′-GGAGCGAGATCCCTCCAAA-3′
		reverse 5′-GGCTGTTGTCATACTTCTCAT-3′
	IL-6	forward 5′-CAATAACCACCCCTGACCCA-3′
		reverse 5′-TCTGAGGTGCCCATGCTACA-3′
	IL-10	forward 5′-CCCTTTGCTATGGTGTCCTT-3′
		reverse 5′-TGGTTTCTCTTCCCAAGACC-3′
	TNF-α	forward 5′-GAGGCCAAGCCCTGGTATG-3′
		reverse 5′-CGGGCCGATTGATCTCAGC-3′
	TGF-β	forward 5′-CTAATGGTGGAAACCCACAA-3′
		reverse 5′-TATCGCCAGGAATTGTTGCT-3′
	A20	forward 5′-TCCTCAGGCTTTGTATTTGA-3′
		reverse 5′-TGTGTATCGGTGCATGGTTTT-3′

RT-PCR was performed with the following conditions: 95 °C for 3 min; 40 cycles: 95 °C for 15 sec, 60 °C for 15 sec, 72 °C for 15 sec; followed by melting curve analysis. The 2^−ΔΔCT^ method was used to quantify the relative expression of these genes.

### Cytokine ELISA

Serum from CIA mice peripheral blood samples and culture supernatant were collected. IL-6 was directly measured with Human IL-6 ELISA Kit (DAKEWE, Shenzhen, China) and Mouse IL-6 ELISA Kit (DAKEWE, Shenzhen, China) according to the manufacturer’s instruction. Optical density was determined with a Bio-Rad Micro-plate reader, and absorption was measured at 450 nm. A standard curve for each measurement was established using a cytokine standard provided by the ELISA Kit. In brief, the samples were added into the antibody-coated 96-well plates and incubated for 90 minutes in 37 °C. After washing for three times, peroxidase-conjugated detection antibody was pipetted into the plates. After two hours of incubation, TMB was added and subsequently stopped after fifteen minutes incubation. Absorbance was read at 450 nm (Epoch, BioTek). The standard curves were generated at the same time.

### Western blot analysis

Protein samples prepared in SDS sample buffer were boiled and separated on 10% SDS-polyacrylamide gel, and then the proteins were transferred to a 0.45 μm poly-vinylidene fluoride blotting membranes. The membranes were then incubated with primary antibodies against the proteins of interest in blocking solution, washed and then incubated with HRP-conjugated secondary antibodies. Finally, an enhanced chemiluminescence substrate was added to the membranes prior to film exposure to detect the proteins according to manufacturer instructions.

### Flow cytometry assay

To assess apoptosis and necroptosis, UC-MSCs, siRNA UC-MSCs and pA20 UC-MSCs were collected and washed in PBS and stained for annexin V and propidium iodide (PI) with the Annexin V-FITC Apoptosis Detection Kit (Millipore, Boston, USA) following the instructions. The cells were analyzed by FACS Calibur flow cytometry (BD Pharmingen, San Diego, USA). The data were processed using FlowJo software.

### Statistical analysis

The experiments were performed independently at least three times. The data were expressed as mean value ± SD. Unpaired and paired t tests or one-way analysis of variance followed by Dunnett’s posttest (for subgroup analyses) was used. All of analyses were conducted using the SPSS 19 statistical software package, and the statistical images were processed by Graph Pad Prism 5.0 software. Bonferroni’s correction was applied for the number of hypotheses made (n), and differences were considered to be statistically significant when p < 0.05.

## Electronic supplementary material

Supplementary Information
