# Supplementary material for: Loss of A20 in BM-MSCs regulates the Th17/Treg balance in Rheumatoid Arthritis
Source: Sci Rep. 2018 Jan 11;8:427. doi: 10.1038/s41598-017-18693-0 (PMC5765124; doi:10.1038/s41598-017-18693-0)

Title:

Loss of A20 in BM-MSCs regulates the Th17/Treg balance in Rheumatoid Arthritis

Authors:

Zhuan Feng,[1,2,3,*] Yue Zhai, [1,2,3,*] Zhaohui Zheng,[1,3,*] Lijie Yang,[4,*] Xing Luo,[1,3,*] Xiwen Dong, [1,2,3] Qing Han,[1,3] Jin Jin, [2,3] Zhi-Nan Chen[2,3] and Ping Zhu[1,3]

[1]Department of Clinical Immunology, Xijing Hospital, The Fourth Military Medical University, No. 127 West Changle Road, Xi'an, Shaanxi Province, People's Republic of China.

[2]Department of Cell Biology, Fourth Military Medical University, China.

[3]National Translational Science Center for Molecular Medicine, Xi'an 710032, China.

[4]Department of hematology, Xijing Hospital, The Fourth Military Medical University, No. 127 West Changle Road, Xi'an, Shaanxi Province, People's Republic of China.

[*]These authors contributed equally to this work.

Correspondence and requests for materials should be addressed to Ping Zhu (email: zhuping@fmmu.edu.cn) or Zhi-Nan Chen (email: znchen@fmmu.edu.cn)

Competing financial interests: The authors declare no competing financial interests.

Supplementary Figure 1. Full length pictures of the blots presented in main figures.

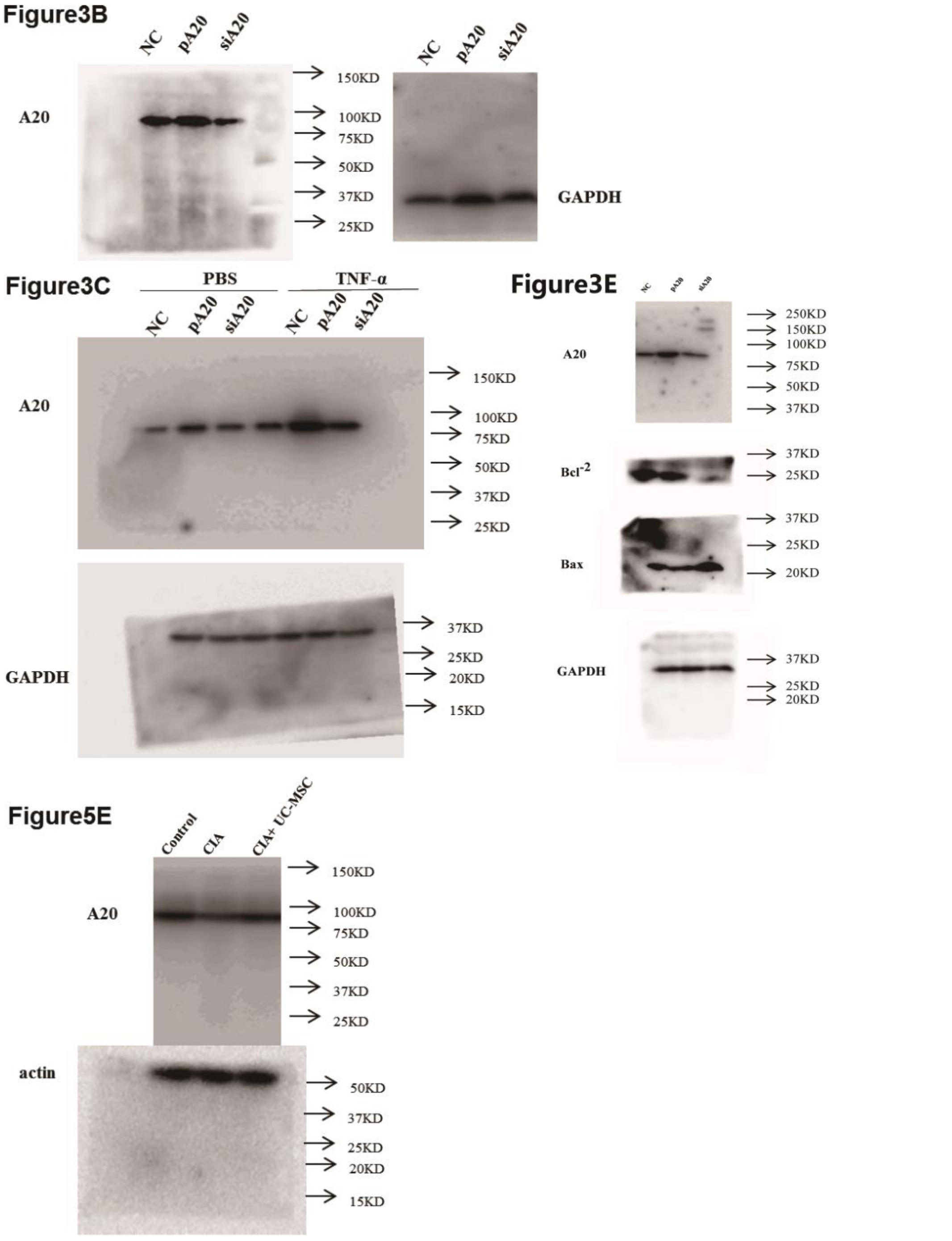

Supplementary Figure 2.

Figure 2B A20 transcription has been shown to be up-regulated in IL-6 and IFN-γ treatment besides TNF-α stimuli, but they don't have a dose-dependent effect on A20 expression.

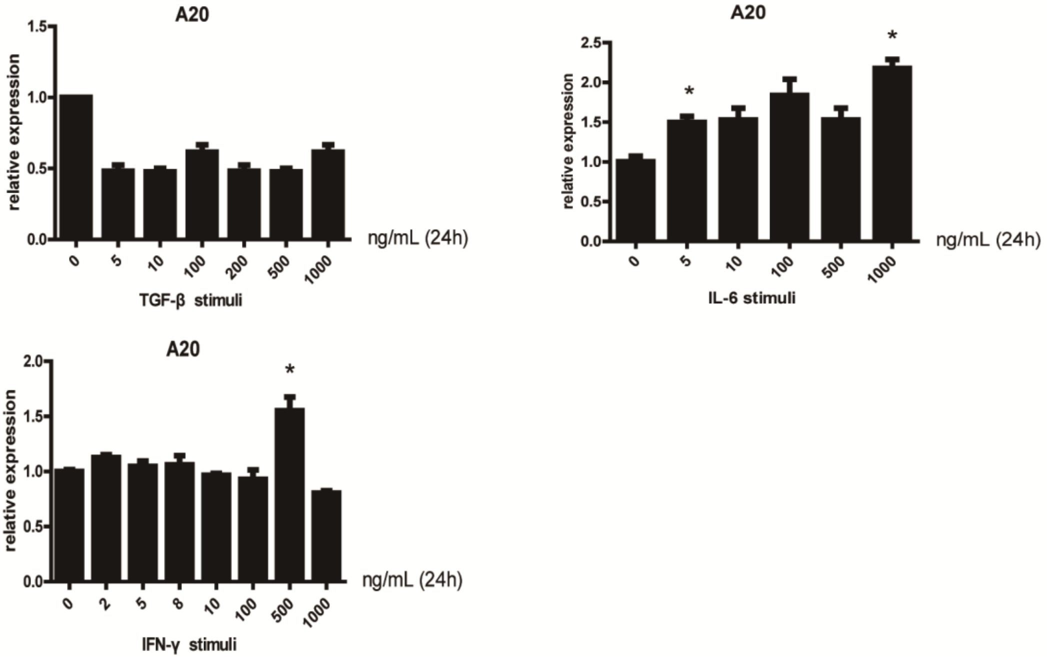

Supplement: Supplementary file 1 — Supplementary Information [file 41598_2017_18693_MOESM1_ESM.pdf]
